# Remission of Invasive, Cancer Stem-Like Glioblastoma Xenografts Using Lentiviral Vector-Mediated Suicide Gene Therapy

**DOI:** 10.1371/journal.pone.0006314

**Published:** 2009-07-20

**Authors:** Peter C. Huszthy, Tsanan Giroglou, Oleg Tsinkalovsky, Philipp Euskirchen, Kai Ove Skaftnesmo, Rolf Bjerkvig, Dorothee von Laer, Hrvoje Miletic

**Affiliations:** 1 Department of Biomedicine, University of Bergen, Bergen, Norway; 2 Georg-Speyer-Haus, Frankfurt am Main, Germany; 3 The Gades Institute, Section for Pathology, Haukeland University Hospital, Bergen, Norway; 4 NorLux Neuro-Oncology Laboratory, CRP-Santé, Luxembourg, Luxembourg; Institute of Cancer Research, United Kingdom

## Abstract

**Background:**

Glioblastoma is the most frequent and most malignant primary brain tumor with a poor prognosis. The translation of therapeutic strategies for glioblastoma from the experimental phase into the clinic has been limited by insufficient animal models, which lack important features of human tumors. Lentiviral gene therapy is an attractive therapeutic option for human glioblastoma, which we validated in a clinically relevant animal model.

**Methodology/Principal Findings:**

We used a rodent xenograft model that recapitulates the invasive and angiogenic features of human glioblastoma to analyze the transduction pattern and therapeutic efficacy of lentiviral pseudotyped vectors. Both, lymphocytic choriomeningitis virus glycoprotein (LCMV-GP) and vesicular stomatitis virus glycoprotein (VSV-G) pseudotyped lentiviral vectors very efficiently transduced human glioblastoma cells *in vitro* and *in vivo*. In contrast, pseudotyped gammaretroviral vectors, similar to those evaluated for clinical therapy of glioblastoma, showed inefficient gene transfer *in vitro* and *in vivo*. Both pseudotyped lentiviral vectors transduced cancer stem-like cells characterized by their CD133-, nestin- and SOX2-expression, the ability to form spheroids in neural stem cell medium and to express astrocytic and neuronal differentiation markers under serum conditions. In a therapeutic approach using the suicide gene herpes simplex virus thymidine kinase (HSV-1*-tk*) fused to eGFP, both lentiviral vectors mediated a complete remission of solid tumors as seen on MRI resulting in a highly significant survival benefit (p<0.001) compared to control groups. In all recurrent tumors, surviving eGFP-positive tumor cells were found, advocating prodrug application for several cycles to even enhance and prolong the therapeutic effect.

**Conclusions/Significance:**

In conclusion, lentiviral pseudotyped vectors are promising candidates for gene therapy of glioma in patients. The inefficient gene delivery by gammaretroviral vectors is in line with the results obtained in clinical therapy for GBM and thus confirms the high reproducibility of the invasive glioma animal model for translational research.

## Introduction

Glioblastoma is the most frequent and most malignant primary brain tumor. Despite advances in neurosurgery, radiation and chemotherapy, the prognosis of patients remains poor with a median survival of 14 months [1].

A major drawback in translational brain cancer research has been the lack of suitable animal models. Syngeneic- or xenograft tumors based on glioblastoma cell lines cultured as monolayers grow as circumscribed and highly angiogenic lesions *in vivo* [2], lacking the invasive tumor cells, which represent an important feature of human glioblastoma. The invasive cells migrate away from the initial tumor mass and can cause recurrent tumors in different regions of the brain. Thus, these cells represent a major therapeutic target.

A recently established model in which glioblastoma biopsy-based spheroids are serially passaged in the brains of nude rats shows highly invasive and angiogenic features [3]. Therefore, this model is well suited for the study of new therapeutic strategies. Still, reports using this or other clinically relevant models for experimental therapy are scarce. Recently, we analyzed the therapeutic potential of the HSV-1-based oncolytic Herpes vector G207 in the biopsy spheroid-based GBM model. The tumor volume in treated animals was reduced compared to control groups, but there was no significant survival advantage [4]. In contrast, the same therapy was more effective in a cell line- based animal model [5] and as a result is currently investigated in a phase I/II clinical study [6]. In the present investigation we used the invasive xenograft model to evaluate transduction and therapeutic efficacy of lentiviral pseudotyped vectors.

Gammaretroviral vectors derived from the Moloney murine leukemia virus (MMLV) have been the most frequently used retroviruses for gene therapy of brain tumors [7]–[10]. However, despite promising results in animal models, clinical trials using retroviral vector supernatants or retroviral packaging cells have failed [11]–[13]. One major drawback of gammaretroviral vectors is the exclusive transduction of dividing cells, since in human gliomas, the majority of tumor cells do not divide within a given treatment window. Therefore, lentiviral vectors with their ability to also transduce non-dividing cells are attractive candidates for the treatment of brain cancer. In previous studies, we have developed gammaretroviral and lentiviral vectors pseudotyped with the glycoproteins (GP) of the lymphocytic choriomeningitis virus (LCMV) [14], [15]. These vectors have a broad host range and can be concentrated by ultracentrifugation for *in vivo* applications. In addition, LCMV-GP is not cytotoxic, and stable recombinant packaging cell lines can be established [16], [17]. Recently, we showed that lentiviral LCMV-GP pseudotypes efficiently delivered transgenes to rat glioma cells *in vivo*, while resident neurons were not transduced [18]. Furthermore, we showed a significant therapeutic effect of LCMV-GP pseudotyped lentiviral vectors in the cell-line based 9L rat glioma model using the suicide gene HSV-1-*tk*. VSV-G lentiviral pseudotypes also showed a significant efficacy, similar to that of LCMV pseudotypes, which was mainly mediated by a bystander effect of transduced normal brain cells [19].

In the presented work, we showed that both, VSV-G and LCMV-GP pseudotyped lentiviruses efficiently transduced human glioma cells *in vitro* and *in vivo*, whereas gammaretroviral transduction was inefficient. The gene transfer to glioma cells was efficient for both lentiviral pseudotyped vector types. However, it was more specific using LCMV-GP pseudotyped vectors, as VSV-G pseudotypes also transduced host brain cells in invasive areas. Analysis of transduced tumor cells revealed that both lentiviral vectors targeted CD133-positive as well as CD133-negative cancer cells. Furthermore, transduced glioblastoma cells expressed the stem cell markers nestin and SOX2. Importantly, when evaluated for therapeutic application using HSV-1-*tk* as a transgene, both lentiviral vectors mediated complete tumor regression on MRI, resulting in a highly significant survival benefit (p<0.001) compared to the control groups.

## Materials and Methods

### Ethics Statement

The collection of human biopsy tissue was approved by the regional ethical committee. The handling of the animals and the surgical procedures were done in accordance with the Norwegian Animal Act and the local ethical committee approved the protocol.

### Cell lines

The human embryonic kidney cell line 293T (ATCC number CRL-11268) and the TE671 cell line were obtained from the American Type Culture Collection (ATCC, Manassas, VA) and maintained in Dulbbeco's modified eagle medium (DMEM) supplemented with 10% fetal calf serum (FCS) and 1% glutamine. All cell lines were grown at 37°C in a humidified atmosphere of 5% CO_2_.

### Tissue culture

Tumor fragments from glioblastoma multiforme patients were obtained during surgery. Tissue specimens were taken from viable tumor areas that corresponded to regions with contrast enhancement on preoperative MRI-scans. The specimens were transferred to test tubes containing complete growth medium, and spheroids were prepared as previously described [20]. The same method was applied for tumor material passaged in nude rats. Briefly, tissue samples were minced into ∼0.5 mm fragments and placed into 80 cm^2^ tissue culture flasks (Nunc, Roskilde, Denmark) base-coated with 0.75% agar (Difco, Detroit, MI). The spheroids were maintained in a standard tissue culture incubator with 5% CO_2_ and 100% relative humidity at 37°C. The medium was changed once a week. Spheroids with diameters between 400 and 600 µm were selected for *in vitro* experiments and for intracerebral implantation.

### Dissociation of tumors

Tumors were dissociated using the Neuronal Dissociation Kit (Miltenyi, Bergisch-Gladbach, Germany) according to the manufacturer's protocol.

### Flow cytometric analysis and cell sorting

Cells were analyzed and sorted on a MoFlo cell sorter (Beckman Coulter, USA; former Cytomation, USA), equipped with a Coherent Enterprise 621 argon-ion laser tuned to 488 nm (used at 180 mW), and 635 nm Diode (25 mW). Two µg/ml propidium iodide – PI (Molecular Probes) were added to the samples before flow sorting to facilitate dead cell discrimination. The GFP and PI were excited at 488 nm and fluorescence was measured through 530/40 BP and 613/20 BP optical filters (all filters from Omega Optical, Brattleboro, VT, USA), respectively. Doublets were discriminated using a forward light scatter (FSC) versus pulse width. FL3 channel (in logarithmic mode) with FSC were used to display and gate out PI positive/dead cells. FSC and side light scatter (SSC) signals were detected and shown in linear mode. GFP+ cells were defined on SSC versus FL1 (in logarithmic mode) dot plot after exclusion of dead cells and debris as described above.

For analysis of CD133 expression cells were stained with allophycocyanin (APC) conjugated monoclonal CD133/1 (AC133) antibodies (Miltenyi, Bergisch-Gladbach, Germany), according to the manufacturer's general protocol for immunofluorescent staining (for 10 min in the dark at 4°C). CD133-APC was excited at 635 nm, the fluorescence was measured through 670/30 BP optical filter, and alive CD133+ cells were defined on SSC versus FL6 (in logarithmic mode) dot plot. Non-stained cell suspension was used as a control.

GFP+ tumor cells were sorted in “purify 1” mode into polypropylene round-bottom Falcon tubes (Becton Dickinson Labware Europe, France) containing culture media, that were placed on ice. Aliquots from some samples at the end of the sorting were removed and reanalyzed for control of the sort purity that was greater than 98%.

### Culture of sorted cells

Sorted cells were either cultured in neurobasal medium (Invitrogen, Carlsbad, CA) with B27 supplement (20 µl/ml; Invitrogen), Glutamax (10 µl/ml; Invitrogen), fibroblast growth factor 2 (20 ng/ml; Peprotech, Rocky Hill, NJ), epidermal growth factor (20 ng ml; Peprotech) or transferred to DMEM supplemented with 10% fetal calf serum (FCS) and 1% glutamine and grown on cover slips in 24 well plates.

### Immunofluorescence staining of spheroids/adherent cells

Spheroids/adherent cells were stained with human-specific mouse-anti-nestin antibodies (Millipore, Billerica, MA), goat-anti-SOX2 antibodies (R&D), mouse-anti-β−tubulinIII (Millipore) antibodies and mouse-anti-GFAP antibodies (Millipore). Primary antibodies were incubated overnight at 4°C. Alexa-Fluor647-goat-anti-mouse und Alexa-Fluor647-donkey-anti-goat antibodies (Dianova, Hamburg, Germany) were used as secondary antibodies over night at 4°C (for spheroids) or for 2h at room temperature (for adherent cells). Spheroids were examined under a fluorescence microscope (Nikon, Tokyo, Japan) and adherent cells were analyzed by confocal scanning laser microscopy (Zeiss, Jena, Germany).

### Lentiviral and Retroviral vectors

The lentiviral vector plasmid pRRL.sinCMVeGFPpre was published by Naldini et al. [21]. The construction of the lentiviral vector pRRL.sinCMV-TK/eGFPpre has been described previously. The retroviral vector pMP71-eGFP-pre has been described previously [22].

### Preparation of lentiviral and retroviral vector supernatants

The 293T cell line was used for transient lentiviral vector production. The lentiviral vector plasmid pRRL.sinCMV-TK/eGFPpre (5 µg) or pRRL.sinCMVeGFPpre (5 µg), the HIV gag-pol-REV expression plasmid pCMV-dR8.91(12.5 µg) [21] and 2 µg of the envelope expression plasmid pHCMV-LCMV-GP [14] or pCMV-G [23] were cotransfected into 293T cells and concentrated as described previously [18]. For the production of retroviral vectors, 293T cells were transfected with 7.5 µg of pMP71-eGFP-pre, 12.5 µg of pSV-Mo-MLVgagpol, and 2 µg of the envelope expression plasmid pHCMV-LCMV-GP [14] or pCMV-G [23]. Vectors were harvested and concentrated as described previously [24].

### Titration of viral vector supernatants

Vectors were titered on TE671 cells as described previously [18].

### Implantation of glioblastoma spheroids

Intracranial implantation of glioblastoma spheroids was done as described previously [25].

### Vector infusion

Three weeks to one month after implantation, the animals were anesthetized and prepared for vector injection. The skin was withdrawn to reveal the location of the craniotomy. 2 times 10 µL of vector stocks were delivered into the centre of the tumors using a glass syringe (model 701, Hamilton, Bonaduz, Switzerland) secured in a microprocessor-controlled infusion pump (UMP 2–1, World Precision Instruments, Aston, Stevenage, UK). The injection coordinates were estimated after analyzing MRI images for each individual lesion. Vector infusion was done by convection enhanced delivery in the course of 25 min (200 nl/min for 10 min, followed by 400 nl/min for 10 min, and finally 800 nl/min for 5 min). After infusion, the needle was left in place for 5 min to avoid vector reflux. The needle was slowly retracted and the skinfolds were closed with polyamide surgical thread. Following surgery, rats were allowed to recover in an incubator set at 35°C before returning them to the cages.

### Treatment of rat gliomas

Rats bearing glioblastoma xenografts were treated by daily i.p. injections of 50 mg/kg ganciclovir (GCV, Roche, Basel, Switzerland).

### Analysis of rat brains

Animals were euthanized and perfused with sterile saline and thereafter with 4% paraformaldehyde. Brains were removed, suspended in 30% sucrose for three days, and then snap frozen in isopentane chilled with dry ice. Coronal sections (12 µm) were prepared on a cryostat. For immunofluorescence analysis, sections were stained with human-specific anti-nestin antibodies (Millipore) for human glioblastoma cells, mouse-anti-NeuN (Millipore) antibodies for neurons, rat specific mouse-anti-nestin antibodies (Millipore) for astrocytes and progenitor cells. Primary antibodies (dilution 1∶200) were incubated overnight at 4°C. Biotinylated goat-anti-mouse and goat–anti-rabbit (Vector Laboratories, Burlinghame, CA) were used as secondary antibodies (dilution 1∶100) for 2 h at room temperature. Sections were incubated with Extravidin-Cy3 (Sigma, St. Louis, MO) as fluorochrome (dilution 1∶200) for 1 h at room temperature. The sections were examined under a fluorescence microscope (Nikon) and analyzed by confocal scanning laser microscopy (Zeiss, Jena, Germany).

For analysis of transduction efficacy, consecutive sections (every 20.-30.) throughout the tumors were examined under a fluorescence microscope (Nikon) with an automated stage using 10×magnification. The transduction volume was calculated using Nikon Lucia imaging software.

### Immunostaining of paraffin sections

Paraffin embedded formalin-fixed tissue sections from rat brain and patient material were placed in xylene bath for 2×3 minutes, absolute ethanol 2×3 minutes, 96% ethanol 2×2 minutes and finally in distilled water for 30 seconds for removal of paraffin and rehydration. Epitope retrieval was performed by heating the sections at 99°C for 20 minutes in 10 mM citrate buffer at pH 6.0. The sections were incubated with a monoclonal human-specific anti-nestin antibody 1∶200 in TBS/1%BSA over night at 4°C. A biotinylated goat-anti-mouse antibody (Vector Laboratories) was used as secondary antibody (dilution 1∶100) for 1 h at room temperature followed by ABC-complex incubation for 30 min. Sections were developed with with 3′3-diaminobenzidine (DAKO Cytomation), following the manufacturer's instructions.

### Magnetic resonance imaging

Using a Bruker Pharmascan 7 Tesla MR scanner (Bruker Biospin, Billerica, MA), axial T2-weighted RARE sequences were acquired (repetition time, 4,200 ms; echo time, 36 ms; slice thickness, 1 mm; field of view, 3.2 cm; matrix size, 256×256; 20 slices). During scanning, the animals were kept under anesthesia with 1.5% isofluorane (Schering-Plough, Kenilworth, NJ) mixed with 50% air and 50% O2.

### Statistical analysis

Survival was analyzed by a log-rank test based on the Kaplan-Meier test using SPSS software. Differences between pairs of groups were determined by the Student's *t*-test. *P* values<0.05 were considered significant.

## Results

### Lentiviral pseudotyped vectors efficiently transduce glioblastoma spheroids *in vitro*

**Figure 1:**
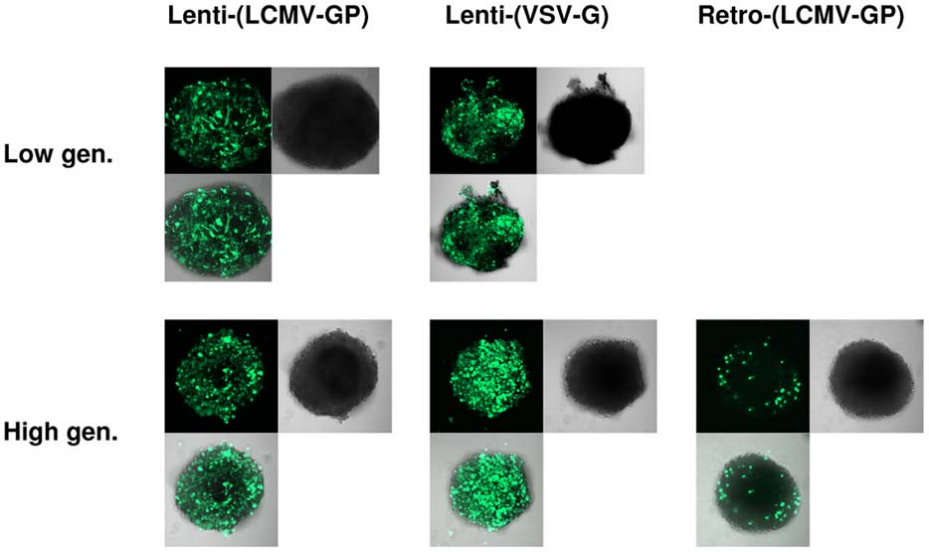
Transduction of glioma spheroids by lenti- and retroviral vectors. Human glioblastoma spheroids derived from a patient biopsy or after serial passaging in nude rats (high generation) were infected by lentiviral vectors pseudotyped with LCMV-GP (5×10^4^) or VSV-G (5×10^4^) or by retroviral vectors pseudotyped with LCMV-GP (1×10^5^). All vectors were expressing the marker gene eGFP. Spheroids were analyzed by confocal microscopy for eGFP expression 7 days after infection. Pictures show eGFP (green), phase contrast and an overlay of both. Low gen.: spheroids directly derived from patient material (low generation). High gen.: spheroids derived from serial *in vivo* passages in the rat brain (high generation). Original magnification 100×.

Human glioblastoma spheroids derived either directly from the patient (low generation) or from serial *in vivo* passages in the brains of nude rats (high generation), were infected with lentiviral LCMV-GP (5×10^4^ in 10 µl) or VSV-G pseudotyped lentiviral vectors (both 5×10^4^ particles in 10 µl) or with retroviral MLV-based vectors pseudotyped with LCMV-GP (1×10^5^ particles in 10 µl). The vectors were prepared in the same way for *in vitro* and *in vivo* experiments (see materials and methods). Both lentiviral vectors transduced patient spheroids and high generation spheroids very efficiently (Figure 1). In contrast, retroviral vectors transduced only a few single cells in high generation spheroids (Figure 1) and failed to transduce patient spheroids (data not shown). In conclusion, both lentiviral vectors are much more efficient in transducing human glioblastoma spheroids *in vitro* than retroviral vectors.

### Lentiviral pseudotyped vectors efficiently and specifically transduce glioblastoma cells *in vivo*

**Figure 2:**
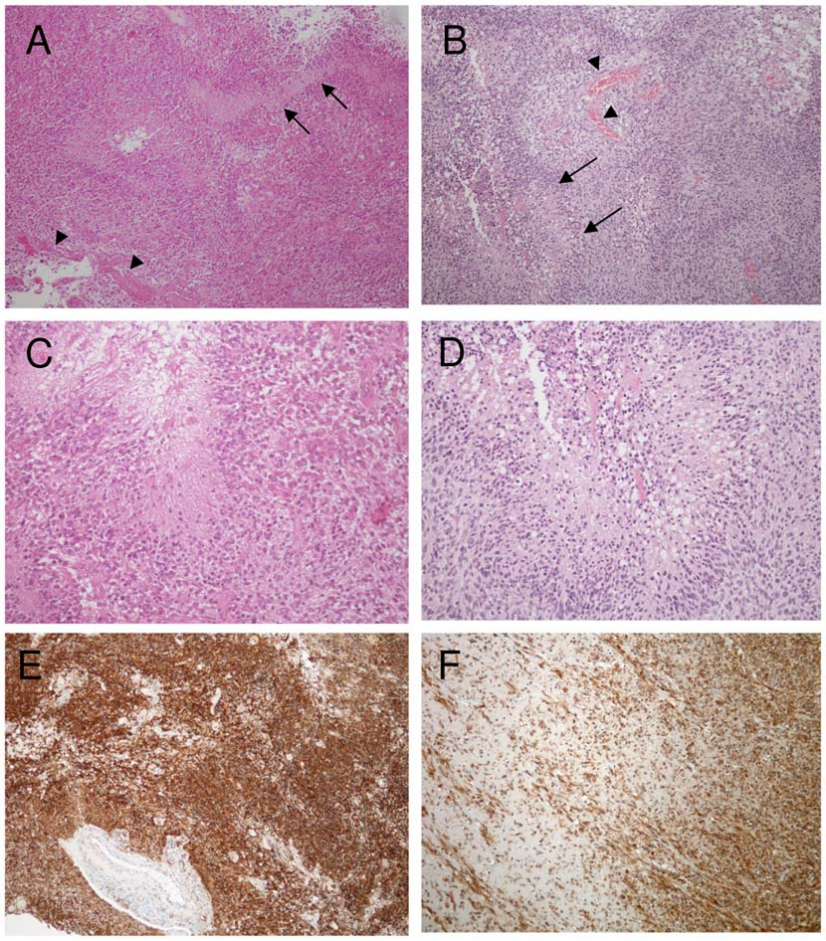
Glioblastoma patient biopsy and xenograft tumor show similar histopathology. Paraffin sections of the patient tumor and the xenograft tumor were stained with H&E (A-D) or immunostained with human-specific anti-nestin antibodies (E,F). Patient tumor (A) and xenograft tumor (B) show angiogenic features of human glioblastoma with palisading necrosis (arrow) and vascular proliferates (arrowheads). Higher magnification of the patient (C) and xenograft tumor (D) demonstrate similar tumor cell morphology with polymorphic nuclei in the vicinity of a tumor necrosis. Patient (E) and xenograft tumor (F) show strong nestin expression of tumor cells. Single tumor cell infiltration into the white matter is observed in the xenograft tumor (F). The patient biopsy was derived only from the solid tumor core. A,B,E,F: Original magnification 100×. C,D: Original magnification 200×.

**Figure 3:**
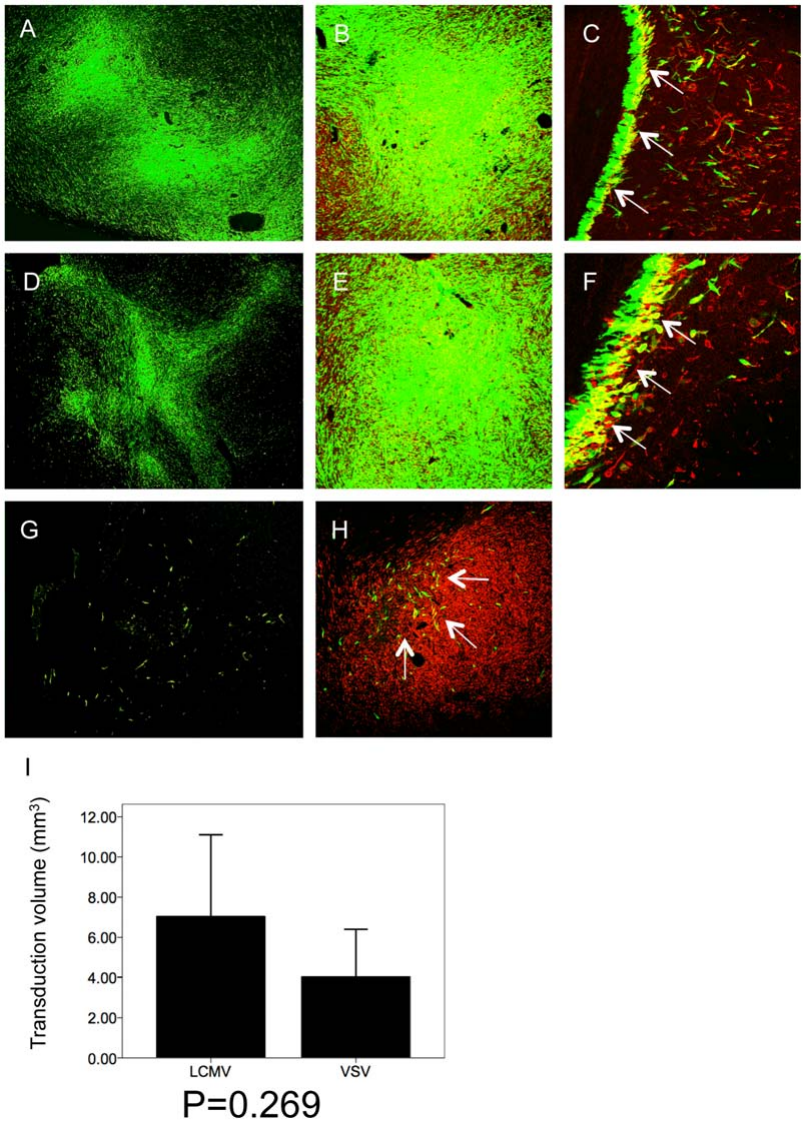
Lentiviral vectors efficiently transduce human glioma cells *in vivo*. Intracranial gliomas were infected with LCMV-GP or VSV-G pseudotyped lentiviral vectors or with LCMV-GP pseudotyped retroviral vectors expressing eGFP 3–4 weeks after spheroid implantation and analyzed by fluorescence (A,D,G) or confocal scanning laser microscopy (B,C,E,F,H) 7 days after infection. The confocal images show overlay of eGFP (green fluorescence) and human-specific nestin (red fluorescence). Tumors were efficiently transduced by lentiviral LCMV-GP (A-C) and VSV-G (D-F) pseudotyped vectors, while retroviral vectors only transduced few scattered tumor cells (G,H; arrows). Transduced glioma cells expressed human-specific nestin in solid (B,E,H) and invasive tumor areas (C,F; arrows). (I) Transduction efficiency of lentiviral vectors was compared quantitatively by measuring the volume of transduction on histological sections using a fluorescence microscope and Nikon imaging software. LCMV transduced tumors (n = 3) showed a higher transduction volume than VSV transduced tumors (n = 3), however, the difference was not statistically significant (p = 0.269). A,D: Original magnification 40×. C,E,G;H: Original magnification 100×. D, F: Original magnification 200×.

To compare the transduction efficiency of lentiviral and gammaretroviral vectors *in vivo*, we used a xenograft model that reflects the angiogenic and invasive features of human glioblastoma *in situ*. The xenograft also expresses the neural progenitor marker nestin and closely recapitulates the histology of the patient tumor (Figure 2). The vectors were injected into the center of progressively growing lesions using microprocessor-controlled stereotactic infusion. The injection coordinates were estimated after analyzing MRI images for each individual lesion. The injection volume applied was 2×10 µl and the vector titre 1×10^7^/ml for all vectors. Transduction efficiency was evaluated 7 days after vector injection. Both lentiviral pseudotyped vectors showed very efficient transduction of the tumors (Figure 3A,D). When analyzed at higher magnification, both LCMV-GP and VSV-G pseudotyped lentiviral vectors showed efficient transgene delivery to nestin-positive tumor cells in solid (Figure 3B,E) and invasive tumor areas (Figure 3C,F). In contrast, the retroviral vector only transduced a few scattered tumor cells near the injection site (Figure 3 G,H). For a quantitative comparison of transduction efficiency between the two lentiviral pseudotyped vectors, the GFP-positive areas were measured on histological slides (see material and methods). The total volume of transduced tumor tissue was 7.05±3.51 mm^3^ for LCMV-GP pseudotyped vectors and 4.05±2.04 mm^3^ for VSV-G pseudotyped vectors (Figure 3I). Although there was a difference in the mean, it was not statistically significant (p = 0.269) due to high interindividual differences (standard deviations).

**Figure 4:**
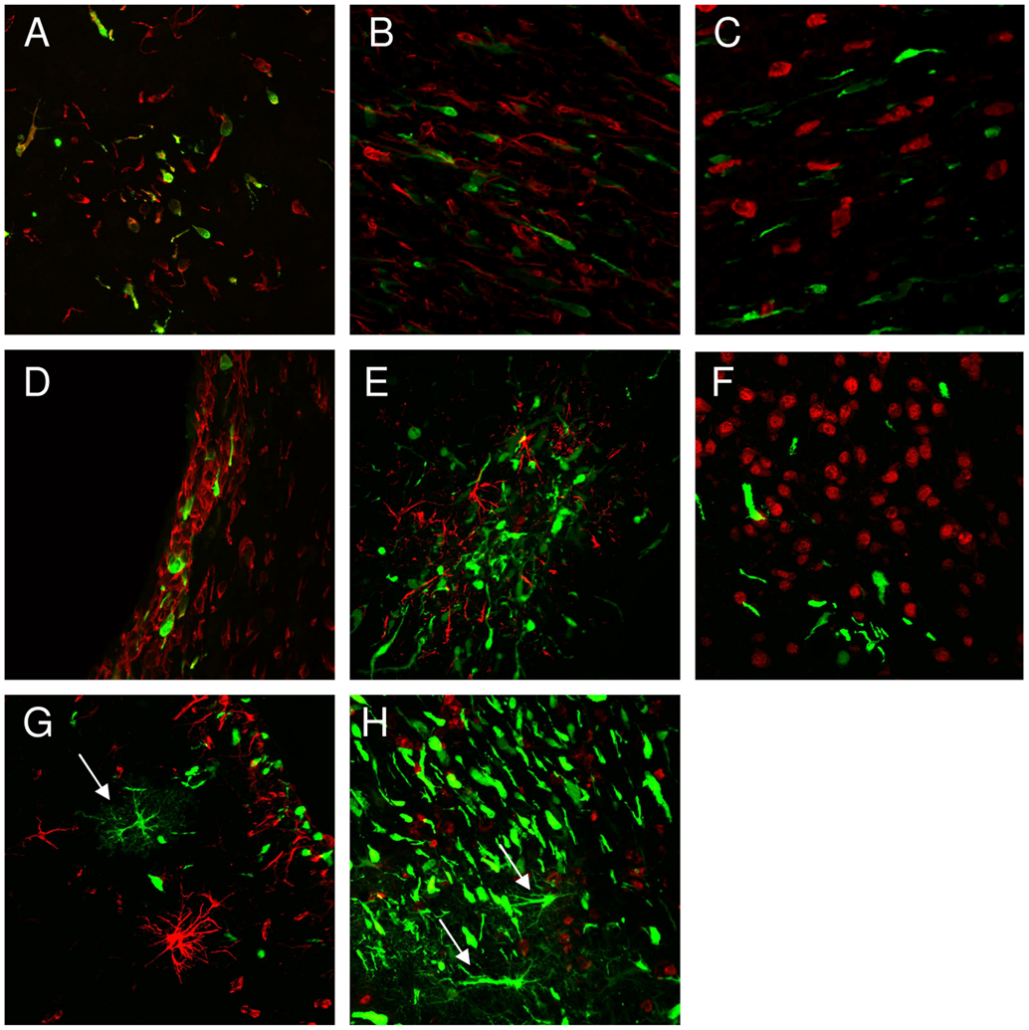
Specificity of tumor cell transduction by lentiviral vectors. Intracranial gliomas were infected with LCMV-GP- or VSV-G-pseudotyped lentiviral vectors expressing eGFP 3–4 weeks after tumor implantation and analyzed confocal laser scanning microscopy 7 days after infection. Transduction of invasive tumor cells was analyzed after staining with human-specific nestin antibodies. Host neurons and astrocytes were labeled using antibodies against NeuN and GFAP. Invasive areas showed single cell invasion by tumor cells (A-C, E, F) or a subependymal accumulation of tumor cells (D,G,H). LCMV pseudotyped vectors specifically transduced invasive glioma cells (A-C). VSV-G pseudotyped vectors showed specific transduction of tumor cells in some invasive areas (D-F), but also transduction of single normal brain cells in others (G,H; arrows). Transduced normal brain cells were mostly detected by morphologic criteria (more processes), as the staining (GFAP or NeuN) not always matched with the transduced cells. A,D: nestin staining, magnification 200×. B,E,G: GFAP staining, magnification 200×. C,F,H: NeuN staining, magnification 200×.

To analyze transduction specificity, histological sections of invasive tumor areas were stained for rat specific markers NeuN (for neurons) and nestin (for astrocytes and progenitor cells). LCMV-GP pseudotyped lentiviral vectors exclusively transduced tumor cells in all invasive areas (Figure 4A), while normal brain cells were not transduced (Figure 4B,C). Also VSV-G pseudotyped vectors showed specific transduction of tumor cells in some invasive areas (Figure 4D,E), however, they also transduced a few host brain cells at other sites (Figure 4G,H).

### Lentiviral pseudotyped vectors transduce cancer-stem-like glioma cells

**Figure 5:**
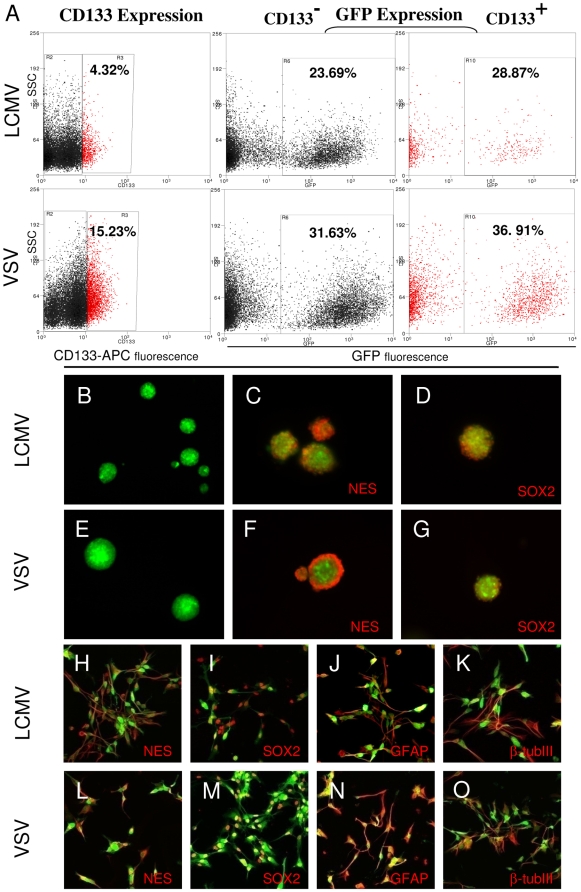
Lentiviral vectors transduce cancer stem-like cells. Intracranial gliomas were infected with LCMV-GP or VSV-G pseudotyped lentiviral vectors expressing eGFP 3–4 weeks after tumor implantation. Tumors were excised when large lesions appeared on MRI and were enzymatically dissociated. The transduction of CD133 positive cells was measured by flow cytometry. (A) LCMV-GP and VSV-G pseudotyped vectors transduce CD133 positive and negative tumor cells. The fraction of transduced (GFP-positive) cells is slightly higher in CD133 positive cells (right column) compared to CD133 negative cells (middle column). GFP+ cells were sorted, cultured in the presence or absence of serum and analyzed by fluorescence (B-G) or confocal microscopy (H-O). LCMV-GP (B) and VSV-G (E) transduced cells form spheroids upon culture in serum-free neural basal medium supplemented with EGF and bFGF. Transduced spheroids express the neural stem cell markers nestin (C,F) and SOX2 (D,G). Transduced cells cultured in serum-containing medium express the stem cell markers nestin (H,L) and SOX2 (I,M), but also the differentiation markers GFAP (J,N) and beta-tubulinIII (K,O). The pictures C,D,E,F,H-O show overlay of the virus-delivered transgene (eGFP, green) and detected antigen (Alexa-647, red).

To analyze the potential of both lentiviral vectors to infect cancer stem-like cells, transduced tumors were enzymatically dissociated and CD133 expression was measured by flow cytometry. Both vectors transduced CD133-positive and CD133-negative cells (Figure 5A). Although there were high interindividual differences in the fraction of total CD133-positive tumor cells in the different xenografts, both vectors showed similar efficiency in transducing CD133-positive cells, which was slightly higher than the overall fraction of CD133-positive cells (table 1).

**Table 1:** Transduction of CD133+ cells by lentiviral vectors.

Virus	Animal	Total CD133+ cells (%)	Transduced CD133+ cells (%) (GFP gated)	Factor* (Increase)
LCMV	1	4.50	5.69	1.264
	2	37.53	41.50	1.106
VSV	1	14.20	16.50	1.162
	2	16.98	20.31	1.196

* Increase in percentage of transduced CD133+ cells compared to total fraction of CD133+ cells.

The GFP-positive cells from tumors transduced with LCMV-GP or VSV-G pseudotyped lentiviral vectors were sorted and cultured in neural basal medium supplemented with EGF and bFGF. Transduced tumor cells from both vectors were able to form spheroids (Figure 5B,E). Spheroids expressed the stem cell markers nestin and SOX2 (Figure 5C,D,F,G). Sorted cells were also plated under serum conditions. The cells continued to show significant expression of the stem cell markers nestin and SOX2, but also of the differentiation markers GFAP and β-tubulinIII (Figure 5H-O).

### Lentiviral pseudotyped vectors expressing the suicide gene HSV-1-*tk* mediate an efficient therapeutic effect *in vivo*

**Figure 6:**
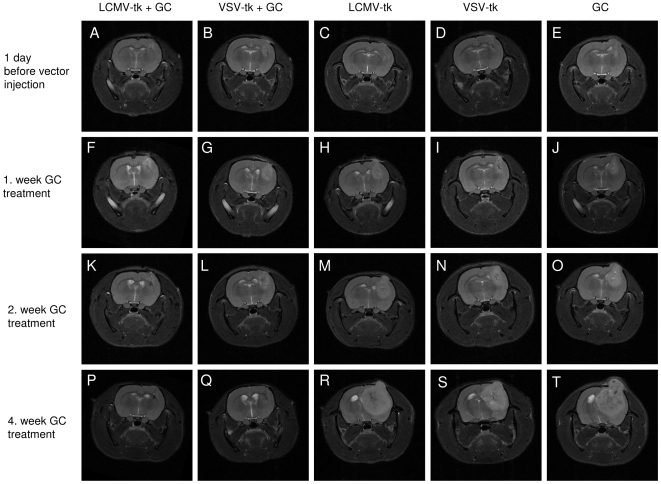
Tumors treated with lentiviral vectors and ganciclovir show complete remission on MRI. Representative three-dimensional MRI (T2 RARE). (A,F,K,P) Lentiviral LCMV-GP vectors with ganciclovir treatment. (B,G,L,Q) Lentiviral VSV-G vectors with ganciclovir treatment. (C,H,M,R) Lentiviral LCMV-GP vectors without ganciclovir treatment. (D,I,N,S) Lentiviral VSV-G vectors without ganciclovir treatment. (E,J,O,T) ganciclovir treatment only. Time points after tumor implantation: (A-E) 1 day before vector injection. (F-J) 1. week ganciclovir treatment. (K-O) 2. week ganciclovir treatment. (P-T) 4. week ganciclovir treatment.

**Figure 7:**
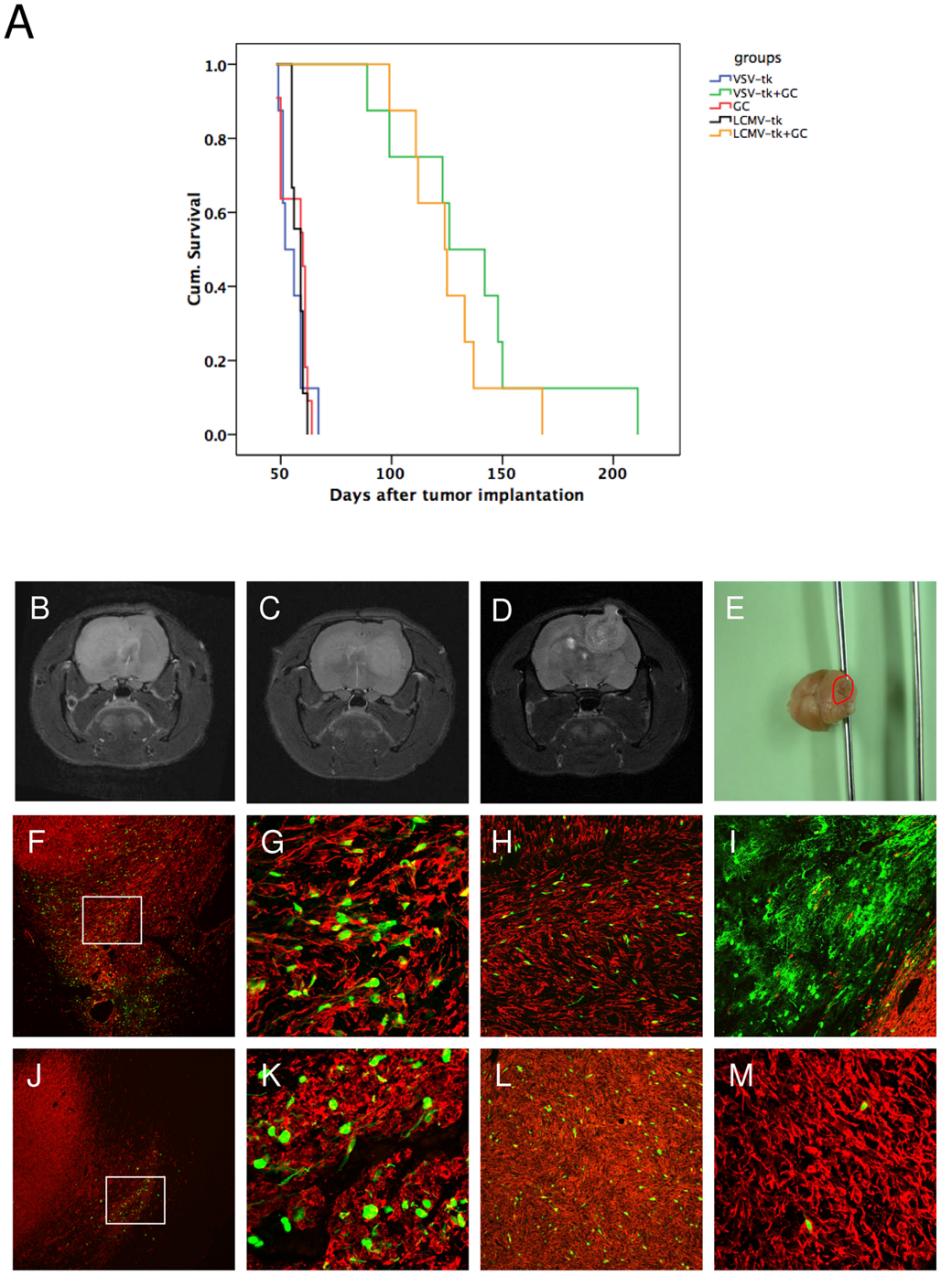
Therapeutic efficiency of LCMV-GP and VSV-G pseudotyped lentiviral vectors *in vivo*. Intracranial gliomas were injected with LCMV-GP or VSV-G pseudotyped lentiviral vectors expressing HSV-1-*tk* fused to eGFP 3 weeks after tumor implantation. 7 days after vector infection, animals in both treated groups and in one control group were treated with ganciclovir for 30 days. (A) Kaplan-Meier survival curve. The survival benefit for both treatment groups compared to control groups was statistically significant (*P*<0.001; log-rank test). There was no significant difference in survival between the two treatment groups. (B-D) Representative MRI (T2 RARE) of recurrent tumors in the LCMV- (B,C) and VSV-treated (D,E) group. (B) Invasive contralateral recurrence. (C) Invasive local and contralateral recurrence. (D) More circumscribed local recurrence. (E) Macroscopic picture of a rat brain with a recurrence in the cerebellum (red circle), treated with VSV-G pseudotyped vectors and GC. (F-M) Sections of recurrent tumors were stained with antibodies against human-specific nestin and analyzed by fluorescence (F,J) or confocal microscopy (G-I,K-M). Pictures show overlay of nestin (red) and eGFP transgene (green). (F-I) Recurrent tumors of animals treated with VSV-G pseudotyped lentiviral vectors. (F) Recurrent tumor with GFP-positive cells in the invasive area. (G) Higher magnification of (F). (H) GFP-positive tumor cells in the corpus callosum region. (I) GFP-positive normal brain cells at the tumor border. (J-M) Recurrent tumors of animals treated with LCMV-GP pseudotyped lentiviral vectors. (J) GFP-positive tumor cells in residual small lesion from the primary tumor. The recurrent tumor is growing from the contralateral hemisphere over the corpus callosum to the ipsilateral hemisphere (arrows). (K) Higher magnification of (J). (L) GFP-positive tumor cells in the solid ipsilateral recurrent lesion. (M) Few GFP-positive cells in a contralateral recurrent tumor. F,J: Magnification 40×. G,K,M: Magnification 200×. H,I,L: Magnification 100×.

To evaluate the therapeutic efficacy of both lentiviral pseudotyoped vectors in the invasive xenograft model, vectors expressing the suicide gene HSV1-*tk* fused to eGFP were injected into established tumors when visible on MRI using the same method as described for the *in vivo* tropism study. The animals were treated daily with 50 mg/kg ganciclovir for 30 days starting 7 days post vector injection. Tumor growth was measured every 7–14 days by MRI. After 4 weeks of treatment, 7 out of 8 animals in both the LCMV- and the VSV-pseudotype treated groups had a complete remission on MRI (Figure 6). One animal in each group had a stable disease until the end of GC treatment. All animals in the control groups developed large tumors during the treatment period of 30 days (Figure 6). Both, LCMV- and VSV-pseudotype treated animals had a highly significant survival advantage (p<0.001) compared to the control groups (Figure 7A). There was no statistically significant difference in survival between the two treatment groups.

Upon cessation of prodrug administration, all animals developed recurrent tumors, which could be classified into three different groups (Figure 7B-E, table 2). The animals showed either 1) local recurrences or 2) local and/or contralateral recurrences or 3) recurrences in other distant brain areas. There was no clear difference in the recurrence pattern between the two vector types, but LCMV-pseudotyped vector-treated animals had more contralateral recurrences, whereas VSV-G pseudotyped vector-treated animals had more local recurrences (Figure 7B-E, table 2). Histopathological and confocal microscopic analysis of the lesions revealed GFP-positive cells in all recurrent tumors demonstrating that not all transduced glioma cells were killed by ganciclovir treatment (Figure 7F-M). In VSV-G pseudotype-treated animals, the GFP-positive surviving cells were found in invasive areas (Figure 7F,G), the corpus callosum region (Figure 7H) and also in some regions of distant recurrences. One animal also showed a focus of transduced normal brain cells at the tumor border that survived GC treatment (Figure 7I). In the LCMV group, most GFP- positive cells were found in the ipsilateral hemisphere, in solid and invasive tumor areas (Figure 7J,K,L), with only a few cells seen in the contralateral hemisphere (Figure 7M).

**Table 2:** Recurrences after gene therapy with lentiviral vectors.

Virus	Local recurrence	Contralateral and/or local recurrence	Other distant recurrence
LCMV	1	7	0
VSV	4	2	2

## Discussion

Future success of glioma gene therapy will depend on more potent vector systems that show higher transduction efficiency than the systems that are available today. In addition, the application of representative animal models that recapitulate both, the invasive and angiogenic features of patient tumors, is vital in order to minimize the huge discrepancies between the experimental results and clinical outcomes previously observed for gene therapeutic strategies for brain cancer.

To this end, we applied one of the most clinically relevant animal models for glioblastoma known. This model was established several years ago [20] and its growth pattern as well as geno- and phenotypic similarity to glioblastoma in patients has been extensively characterized [3]. A striking difference of our model compared to other cell-line based models is the highly invasive behaviour of the lesions, similar to glioblastoma in patients. Our model is based on spheroids derived from patient biopsies that are passaged serially in the brains of nude rats. First generation tumors are highly invasive and grow without signs of angiogenesis. Late generation tumors show an angiogenic phenotype, but are still invasive. Our *in vitro* experiments revealed that both lentiviral vectors transduced spheroids derived from both low and high generation tumors very efficiently. In contrast, retroviral vectors transduced only high generation spheroids and displayed a much lower efficiency of gene transfer than both lentiviral vectors. This difference can only be attributed to the vector backbone, as the glycoprotein which is responsible for virus entry into the cell was the same for the retroviral and one of the lentivral vectors applied (LCMV-GP). The most important feature that distinguishes lentiviral from retroviral vectors is their ability to infect non-dividing cells. It is known that glioma spheroids, especially primary biopsy spheroids, contain a significant fraction of non-dividing cells, which cannot be transduced by retroviral vectors. It has previously been shown that the cultured biopsy spheroids show a similar cell proliferation as seen in glioblastoma patients [20].

Previous studies have demonstrated that retroviral vectors can very efficiently transduce highly proliferative monolayer cultures of glioma cell lines [14]. However, monolayer cultures change their geno- and phenotypic characteristics under long term culture and thus are not a suitable model to answer clinically relevant questions [26].

For *in vivo* experiments, we selected a high generation xenograft that showed both angiogenic and invasive features and recapitulated the histology of the patient lesion. Furthermore, this xenograft showed a high level of nestin expression similar to the patient material. In translational research it is crucial to assure that the experimental tumors truly reflect the corresponding patient's tumor properties to avoid using non-relevant animal models. However, this strategy is not common practice yet, based on the simplicity of using established cell lines for *in vivo* experiments.

We showed a high transduction efficiency of lentiviral vectors for glioma cells *in vivo*, whereas retroviral vectors transduced a few scattered tumor cells near the injection track. This is in contrast to *in vivo* studies by others where retroviral vectors were very efficient, but as mentioned above, the models applied were non-invasive, based on cell lines cultured as monolayers [27]. Of note, the results of clinical studies using retroviral vectors showed the same low transduction efficiency as observed in our model system [12]. This finding provides further evidence that the glioma model used here has a higher predictive value for the performance of a novel therapeutic approach in the clinic than previous animal models.

The tropism for glioma cells was more specific with LCMV-GP lentiviral pseudotyped vectors, as VSV-G pseudotyped lentiviral vectors also transduced few normal brain cells in invasive areas. In previous studies using a rat glioma model, we also showed a more specific transduction of glioma cells by LCMV-GP pseudotyped vectors compared to VSV-G pseudotyped vectors [18], [19]. In fact, in these studies, the VSV-G pseudotyped vectors transduced normal brain cells at a much higher frequency than in this study. This can be explained by the mode of vector delivery, because in the previous studies, we injected the vectors both into the tumor core as well as into tumor border areas. In the present study, we injected the vectors into the tumor core only using convection enhanced delivery. We used this method because it results in a high distribution volume of drug and vector and is currently used as a delivery method in clinical studies [28]. In addition, the tumor model we apply here is highly invasive and lacks a sharply demarcated brain tumor/normal brain interface, present in the rat glioma model. Another explanation could be the species difference as we used human glioma cells in this study in contrast to rat glioma cells in the previous work. VSV-G pseudotyped vectors might have a higher tropism for human glioma cells than for rat normal host cells. However, as the receptor for VSV-G is unknown [29], this remains a hypothesis.

The targeting of cancer stem cells or cancer stem-like cells in human tumors including glioblastoma has recently evolved as a major aim in cancer therapy. These stem cells are suggested to initiate cancer and might be resistant to therapy, thus being responsible for tumor recurrence [30]–[32]. Yet, recent studies have initiated a controversial discussion whether cancer stem cells really exist [33], [34]. Therefore, we use the term “cancer stem-like cells” in our study to designate cells which have certain stem-like properties described previously. We showed that both lentiviral vectors transduced CD133-positive and CD133-negative cells. CD133-positive cells have been identified as cancer initiating cells and cancer stem cells in many different cancers including glioblastoma [30], [35]. However, more recent reports questioned these findings showing that CD133-negative popopulations can include cancer initiating cells as well [36], [37]. The efficient targeting of CD133-positive and CD133-negative glioma cells has also been described for adenoviral vectors [38], [39].

We further demonstrated that sorted cells from tumors transduced either by lentiviral LCMV-GP or VSV-G pseudotyped lentiviral vectors had the ability to form spheroids upon culturing in neural basal medium supplemented with EGF and bFGF. Spheroids from both sorted cell populations expressed the neural stem cell markers nestin and SOX2 and showed the ability to express the differentiation markers GFAP and β-tubulinIII under serum conditions. These properties have been described for neural progenitor cells [40] as well as for cancer stem-like cells in human glioblastoma [26]. Thus, the cell populations transduced by LCMV or VSV pseudotyped lentiviral vectors showed features of cancer stem-like cells which might be an important target for therapy.

In a therapeutic approach using the suicide gene HSV-1*-tk* fused to eGFP, we demonstrated a highly significant therapeutic effect for both lentiviral vectors compared to control groups. Using MRI to follow tumor growth, we detected complete remission in 7 out of 8 animals for LCMV-GP and VSV-G pseudotyped vectors after 30 days of ganciclovir treatment. HSV-1-*tk* has been reported to be an effective therapeutic gene in previous studies [19], [41]. The limited success in clinical studies has been a result of inefficient gene delivery systems rather than lack of efficacy of the suicide mechanism [11]. However, there is still space for improvement of the prodrug delivery such as application length, treatment intervals and route of delivery. Tai et al. demonstrated that multiple cycles of prodrug application are superior over one cycle of prodrug [42]. In our study, we still detected GFP-positive tumor cells after one cycle (30 days) of ganciclovir administration in treated animals indicating that application in cycles might also improve the therapeutic effect in this setting. Further, these results demonstrate that vector-transduced tumor cells retain the ability to invade brain tissue and migrate even to distant brain regions.

In conclusion, the present study demonstrates an efficient transduction and therapy of experimental human glioblastoma by lentiviral vectors. The inefficient gene transfer by gammaretroviral vectors is in line with the results obtained in clinical trials and thus confirms the high relevance of the spheroid-based glioma animal model for translational research.

## References

[1] Stupp R, Mason WP, van den Bent MJ, Weller M, Fisher B (2005). Radiotherapy plus concomitant and adjuvant temozolomide for glioblastoma.. N Engl J Med.

[2] Mahesparan R, Read TA, Lund-Johansen M, Skaftnesmo KO, Bjerkvig R (2003). Expression of extracellular matrix components in a highly infiltrative *in vivo* glioma model.. Acta Neuropathol.

[3] Sakariassen PO, Prestegarden L, Wang J, Skaftnesmo KO, Mahesparan R (2006). Angiogenesis-independent tumor growth mediated by stem-like cancer cells.. Proc Natl Acad Sci U S A.

[4] Huszthy PC, Goplen D, Thorsen F, Immervoll H, Wang J (2008). Oncolytic herpes simplex virus type-1 therapy in a highly infiltrative animal model of human glioblastoma.. Clin Cancer Res.

[5] Mineta T, Rabkin SD, Yazaki T, Hunter WD, Martuza RL (1995). Attenuated multi-mutated herpes simplex virus-1 for the treatment of malignant gliomas.. Nat Med.

[6] Markert JM, Liechty PG, Wang W, Gaston S, Braz E (2009). Phase Ib trial of mutant herpes simplex virus G207 inoculated pre-and post-tumor resection for recurrent GBM.. Mol Ther.

[7] Benedetti S, Bruzzone MG, Pollo B, DiMeco F, Magrassi L (1999). Eradication of rat malignant gliomas by retroviral-mediated, *in vivo* delivery of the interleukin 4 gene.. Cancer Res.

[8] Kunkel P, Ulbricht U, Bohlen P, Brockmann MA, Fillbrandt R (2001). Inhibition of glioma angiogenesis and growth *in vivo* by systemic treatment with a monoclonal antibody against vascular endothelial growth factor receptor-2.. Cancer Res.

[9] Tamura K, Tamura M, Ikenaka K, Yoshimatsu T, Miyao Y (2001). Eradication of murine brain tumors by direct inoculation of concentrated high titer-recombinant retrovirus harboring the herpes simplex virus thymidine kinase gene.. Gene Ther.

[10] Wang WJ, Tai CK, Kasahara N, Chen TC (2003). Highly efficient and tumor-restricted gene transfer to malignant gliomas by replication-competent retroviral vectors.. Hum Gene Ther.

[11] Rainov NG, Ren H (2003). Clinical trials with retrovirus mediated gene therapy–what have we learned?. J Neurooncol.

[12] Rainov NG (2000). A phase III clinical evaluation of herpes simplex virus type 1 thymidine kinase and ganciclovir gene therapy as an adjuvant to surgical resection and radiation in adults with previously untreated glioblastoma multiforme.. Hum Gene Ther.

[13] Sandmair AM, Loimas S, Puranen P, Immonen A, Kossila M (2000). Thymidine kinase gene therapy for human malignant glioma, using replication-deficient retroviruses or adenoviruses.. Hum Gene Ther.

[14] Beyer WR, Westphal M, Ostertag W, von Laer D (2002). Oncoretrovirus and lentivirus vectors pseudotyped with lymphocytic choriomeningitis virus glycoprotein: generation, concentration, and broad host range.. J Virol.

[15] Miletic H, Bruns M, Tsiakas K, Vogt B, Rezai R (1999). Retroviral vectors pseudotyped with lymphocytic choriomeningitis virus.. J Virol.

[16] Beyer WR, Miletic H, Ostertag W, von Laer D (2001). Recombinant expression of lymphocytic choriomeningitis virus strain WE glycoproteins: a single amino acid makes the difference.. J Virol.

[17] Fischer YH, Miletic H, Giroglou T, Litwak S, Stenzel W (2007). A retroviral packaging cell line for pseudotype vectors based on glioma-infiltrating progenitor cells.. J Gene Med.

[18] Miletic H, Fischer YH, Neumann H, Hans V, Stenzel W (2004). Selective transduction of malignant glioma by lentiviral vectors pseudotyped with lymphocytic choriomeningitis virus glycoproteins.. Hum Gene Ther.

[19] Miletic H, Fischer YH, Giroglou T, Rueger MA, Winkeler A (2007). Normal brain cells contribute to the bystander effect in suicide gene therapy of malignant glioma.. Clin Cancer Res.

[20] Bjerkvig R, Tonnesen A, Laerum OD, Backlund EO (1990). Multicellular tumor spheroids from human gliomas maintained in organ culture.. J Neurosurg.

[21] Naldini L, Blomer U, Gallay P, Ory D, Mulligan R (1996). *In vivo* gene delivery and stable transduction of nondividing cells by a lentiviral vector.. Science.

[22] Schambach A, Wodrich H, Hildinger M, Bohne J, Krausslich HG (2000). Context dependence of different modules for posttranscriptional enhancement of gene expression from retroviral vectors.. Mol Ther.

[23] Yee JK, Miyanohara A, LaPorte P, Bouic K, Burns JC (1994). A general method for the generation of high-titer, pantropic retroviral vectors: highly efficient infection of primary hepatocytes.. Proc Natl Acad Sci U S A.

[24] Giroglou T, Cinatl J, Rabenau H, Drosten C, Schwalbe H (2004). Retroviral vectors pseudotyped with severe acute respiratory syndrome coronavirus S protein.. J Virol.

[25] Huszthy PC, Svendsen A, Wilson JM, Kotin RM, Lonning PE (2005). Widespread dispersion of adeno-associated virus serotype 1 and adeno-associated virus serotype 6 vectors in the rat central nervous system and in human glioblastoma multiforme xenografts.. Hum Gene Ther.

[26] Lee J, Kotliarova S, Kotliarov Y, Li A, Su Q (2006). Tumor stem cells derived from glioblastomas cultured in bFGF and EGF more closely mirror the phenotype and genotype of primary tumors than do serum-cultured cell lines.. Cancer Cell.

[27] Galipeau J, Li H, Paquin A, Sicilia F, Karpati G (1999). Vesicular stomatitis virus G pseudotyped retrovector mediates effective *in vivo* suicide gene delivery in experimental brain cancer.. Cancer Res.

[28] Ferguson S, Lesniak MS (2007). Convection enhanced drug delivery of novel therapeutic agents to malignant brain tumors.. Curr Drug Deliv.

[29] Coil DA, Miller AD (2004). Phosphatidylserine is not the cell surface receptor for vesicular stomatitis virus.. J Virol.

[30] Singh SK, Hawkins C, Clarke ID, Squire JA, Bayani J (2004). Identification of human brain tumour initiating cells.. Nature.

[31] Galli R, Binda E, Orfanelli U, Cipelletti B, Gritti A (2004). Isolation and characterization of tumorigenic, stem-like neural precursors from human glioblastoma.. Cancer Res.

[32] Bao S, Wu Q, McLendon RE, Hao Y, Shi Q (2006). Glioma stem cells promote radioresistance by preferential activation of the DNA damage response.. Nature.

[33] Kelly PN, Dakic A, Adams JM, Nutt SL, Strasser A (2007). Tumor growth need not be driven by rare cancer stem cells.. Science.

[34] Quintana E, Shackleton M, Sabel MS, Fullen DR, Johnson TM (2008). Efficient tumour formation by single human melanoma cells.. Nature.

[35] Singh SK, Clarke ID, Terasaki M, Bonn VE, Hawkins C (2003). Identification of a cancer stem cell in human brain tumors.. Cancer Res.

[36] Beier D, Hau P, Proescholdt M, Lohmeier A, Wischhusen J (2007). CD133(+) and CD133(−) glioblastoma-derived cancer stem cells show differential growth characteristics and molecular profiles.. Cancer Res.

[37] Wang J, Sakariassen PO, Tsinkalovsky O, Immervoll H, Boe SO (2008). CD133 negative glioma cells form tumors in nude rats and give rise to CD133 positive cells.. Int J Cancer.

[38] Nandi S, Ulasov IV, Tyler MA, Sugihara AQ, Molinero L (2008). Low-dose radiation enhances survivin-mediated virotherapy against malignant glioma stem cells.. Cancer Res.

[39] Skog J, Edlund K, Bergenheim AT, Wadell G (2007). Adenoviruses 16 and CV23 efficiently transduce human low-passage brain tumor and cancer stem cells.. Mol Ther.

[40] Schwartz PH, Bryant PJ, Fuja TJ, Su H, O'Dowd DK (2003). Isolation and characterization of neural progenitor cells from post-mortem human cortex.. J Neurosci Res.

[41] Miletic H, Fischer Y, Litwak S, Giroglou T, Waerzeggers Y (2007). Bystander killing of malignant glioma by bone marrow-derived tumor-infiltrating progenitor cells expressing a suicide gene.. Mol Ther.

[42] Tai CK, Wang WJ, Chen TC, Kasahara N (2005). Single-shot, multicycle suicide gene therapy by replication-competent retrovirus vectors achieves long-term survival benefit in experimental glioma.. Mol Ther.

